# Association of plasma leptin, pro‐inflammatory adipokines and cancer‐related fatigue in early‐stage breast cancer patients: A prospective cohort study

**DOI:** 10.1111/jcmm.14319

**Published:** 2019-04-23

**Authors:** Yi Long Toh, Chia Jie Tan, Angie Hui Ling Yeo, Maung Shwe, Han Kiat Ho, Yan Xiang Gan, Koon Mian Foo, Pat Chu, Karin Olson, Alexandre Chan

**Affiliations:** ^1^ Department of Pharmacy National University of Singapore Singapore; ^2^ Department of Pharmacy National Cancer Centre Singapore Singapore; ^3^ Department of Pharmacy KK's Women and Children Hospital Singapore; ^4^ Singapore Cord Blood Bank Singapore; ^5^ Faculty of Nursing University of Alberta Edmonton Alberta Canada

**Keywords:** biomarker, breast cancer, cancer‐related fatigue, cytokines, Leptin

## Abstract

Cancer‐related fatigue (CRF) is subjective and has wide inter‐individual variability. Given that leptin is commonly associated with fatigue syndrome, its use as a potential biomarker for CRF is being investigated. The primary objective of this study was to evaluate the association between leptin and CRF in early‐stage breast cancer patients receiving chemotherapy. In a prospective cohort study, patients completed assessments at baseline (T1), during chemotherapy (T2) and after chemotherapy (T3). Levels of plasma leptin and adipokines were measured using a Luminex bead‐immunoassay and CRF was measured using the Multi‐Dimensional Fatigue Symptom Inventory‐Short Form (MFSI‐SF). Data were analysed longitudinally using a generalised estimating equation incorporating clinically relevant parameters and pro‐inflammatory adipokines. The analysis included 136 patients (mean age ± SD = 51.5 ± 8.8 years; 69.1% receiving anthracycline‐based chemotherapy). More patients experienced CRF at T3 (23.8%) than at T2 (13.8%) compared to baseline. An increase was observed in the median plasma leptin level at T2, followed by a decrease at T3 (T1: 4.07 ng/mL, T2: 4.95 ng/mL and T3: 3.96 ng/mL). In the multivariate model, the change in leptin levels over time was significantly associated with the total MFSI‐SF score (*β* = −0.15, *P* = 0.003) after adjusting for the tumour necrosis factor‐α (TNF‐α) level, anxiety, depression, insomnia, age, menopausal status and type of chemotherapy. This is the first study to report leptin as a biomarker that predicts the onset of CRF over time. Future studies are required to validate the findings.

## 1 INTRODUCTION

The estimated incidence of cancer‐related fatigue (CRF) has been reported to range from 28% to 91% among breast cancer patients,^1^ depending on the type of cancer, treatment modality and method of assessment. CRF is characterised by a persistent sense of tiredness that may be related to cancer and/or cancer treatment, is disproportionate to recent activity and is not alleviated by rest.^2^ Left unresolved, CRF adds on to patients' distress and has a negative impact on their quality of life.^3^

Given the extent of the impact of CRF on daily functioning,^4^ addressing CRF should be an integral part of cancer‐supportive care. Nevertheless, this has proved challenging due to the lack of an objective biomarker to monitor these symptoms. The experience of fatigue is subjective in nature and has wide inter‐individual variability because some patients may have poor physical functioning and impaired performance status yet will not complain of fatigue, and vice versa. One study has shown that pro‐inflammatory cytokines could be potentially employed as biomarkers of CRF in patients with advanced cancer,^5^ and that changes from baseline of selected biomarkers were associated with changes in patient‐reported outcome measures of appetite and fatigue. However, the circadian variability and fluctuation of cytokine levels could influence the outcomes associated with the biomarkers used and hinder their utility.^6^ Thus, further research is warranted to determine a suitable biomarker for monitoring CRF and related symptoms in cancer patients.

Leptin is an endocrine hormone that is more commonly known for its metabolic effects, which range from appetite suppression to regulating body weight.^7^ Leptin has shown moderate correlations to varying extents with fatigue in cohorts of patients with cardiovascular risk factors (r = 0.22),^8^ chronic fatigue syndrome (r = −0.51 to 0.73),^6^ chronic hepatitis C infection (r = 0.25; r = 0.30),^9^, ^10^ and irritable bowel syndrome (r = 0.60).^11^ In another study, researchers found that plasma leptin levels could be induced by hydrocortisone, and were markedly increased in patients with chronic fatigue syndrome.^12^ Hence, we hypothesized that leptin levels may be positively correlated with increases in fatigue in cancer patients, as their pro‐inflammatory markers are typically elevated.

This study was designed to investigate the association between plasma leptin levels and CRF in a cohort of early‐stage breast cancer patients, in relation with other relevant clinical factors and adipokines. Our primary objective was to assess leptin's potential to function as a biomarker that could predict the onset of CRF.

## 2 MATERIALS AND METHODS

### 2.1 Study design

This prospective cohort study was conducted between 2014 and 2017 in Singapore. The study was approved by the Singhealth Institutional Review Board (CIRB 2014/754/B), and written informed consent was obtained from all study participants.

### 2.2 Inclusion and exclusion criteria

The inclusion criteria were: (a) diagnosis with early stage (I‐III) breast cancer; (b) no prior history of chemotherapy or radiotherapy; (c) scheduled to receive standard adjuvant chemotherapy; (d) ambulatory status with an Eastern Cooperative Oncology Group score of 0 or 1; and (e) the ability to understand either English or Chinese. Patients were excluded if they were diagnosed with metastatic cancer, had another medical condition that precipitates fatigue (such as severe anemia or thyroid dysfunction), or were on medications such as beta‐blockers that might precipitate fatigue as a side effect.

### 2.3 Study procedures

Patients were assessed at three time points: baseline before treatment initiation (T1), at least 6 weeks after baseline during chemotherapy (T2), and at least 12 weeks after baseline after the completion of chemotherapy (T3). Upon recruitment, patients' demographic information and medication information were collected through patient interviews and electronic databases.

### 2.4 Study tools

At each time point, patients completed a series of questionnaires assessing their fatigue, anxiety, depression and health‐related quality of life (HRQoL). Both English and Chinese versions of all study tools were available and administered by bilingual interviewers. The duration to complete all assessments was approximately 40 minutes. The tools included the following:

1. The Multi‐dimensional Fatigue Symptom Inventory‐Short Form (MFSI‐SF) is questionnaire for measuring fatigue in cancer patients. It consists of five subscales with six items each: general fatigue, physical fatigue, emotional fatigue, mental fatigue and vigour. Each domain is rated on a scale of 0‐4. The total score is obtained by summing all the dimensions except the vigour domain which is subtracted. The total score ranges from −24 to 96, with higher scores indicating more fatigue. We previously established the range of minimal clinically important difference (MCID) for fatigue worsening as 4.50‐10.79.^13^ The psychometric properties and measurement equivalence of the English and Chinese versions of MFSI‐SF have been demonstrated in breast cancer patients in Singapore.^14^
2. The Beck Anxiety Inventory (BAI) is a validated questionnaire that measures the level of anxiety in patients.^15^ It comprises 21 items in which patients rate their somatic, subjective and panic‐related aspects of anxiety on a scale of 0‐3. The summation of the scores gives a total score ranging from 0 to 63, with a higher score indicating a greater anxiety level. Our research team has previously validated the psychometric properties and language equivalence of the English and Chinese versions of BAI.^16^
3. The Beck Depression Inventory (BDI) is a validated questionnaire that assesses the severity of depression^17^ and had been previously used by our team on breast cancer patients.^18^ There are 21 items that patients rate on a scale of 0‐3. The summation of the scores gives a total score ranging from 0 to 63, with a higher score indicating greater severity of depression.
4. The European Organisation for Research and Treatment of Cancer Quality of Life Questionnaire (EORTC QLQ‐C30) assesses HRQoL. This tool was used for the assessment of insomnia in patients, with a higher score on the symptom scale indicating greater severity. Both the English and Chinese versions had been validated for use in cancer patients.^19^, ^20^

### 2.5 Quantification of plasma pro‐inflammatory adipokines and leptin levels

At each time point, 10 ml blood samples were drawn for the analysis of pro‐inflammatory adipokines and kept in an ethylenediaminetetraacetic acid (EDTA) tube. The blood was centrifuged at 1069 x *g* for 10 minutes within 40 minutes of collection and kept frozen at −80°C until analysis. Levels of leptin were quantified using the Human Cancer Biomarker Panel (Biorad, CA, USA) while the levels of IL‐6, IL‐8 and tumour necrosis factor‐α (TNF‐α) were quantified using the Multiplex immunoassay (Luminex™) according to the manufacturer's instructions. IL‐6, IL‐8 and TNF‐α are other known pro‐inflammatory adipokines^21^, ^22^, ^23^ that were quantified so that their relationships with leptin and fatigue could be controlled in the analysis. Each analysis was conducted in duplicate, with an intra‐assay coefficient of variation of ≤10% being considered acceptable.^24^

### 2.6 Statistical analysis

Patients' demographic information, clinical characteristics at baseline and the proportions of patients with CRF were summarised using descriptive statistics. The Friedman test was used to analyse the changes in plasma adipokines and leptin over the various time points, and post hoc Wilcoxon signed‐rank test was used to identify the time points at which a change was observed. Spearman's rank correlation coefficient was used to examine the relationship between each variable and leptin at each time point. Spearman's rank correlation was also used to determine the association between continuous variables of interest and total MFSI‐SF score. The Mann‐Whitney U test was used to compare fatigue levels between groups for categorical variables.

The longitudinal association of CRF with plasma leptin and adipokine levels was assessed using the generalised estimating equation model. The correlation structure with the smallest criterion was chosen using the quasi‐likelihood under the independence model criterion. Leptin was first analysed as a single variable. Next, the following known confounders that affect CRF were analysed individually against total MFSI‐score across the time points: anxiety,^25^ depression,^25^ insomnia,^26^ menopausal status,^27^ anaemia status,^28^ body mass index (BMI),^29^ age,^30^ type of chemotherapy,^31^ and pro‐inflammatory adipokines such as IL‐6, IL‐8 and TNF‐α.^21^, ^22^, ^23^ Confounders with *P*‐values <0.05 were considered to be statistically significant and included in the final model as covariates to examine plasma leptin levels with respect to fatigue levels. All statistical analyses were conducted with STATA version 15 (Statacorp, TX, 2017), and two‐sided *P*‐values <0.05 were considered statistically significant.

## 3 RESULTS

### 3.1 Patient demographics

A total of 136 early‐stage breast cancer patients were included in this analysis, and their demographics are summarised in Table 1. The mean age ± standard deviation (SD) of the patients was 51.5 ± 8.8 years, and the mean BMI ± SD was 23.9 ± 4.1 kg/m^2^. Majority of the patients were Chinese (82.4%), had been diagnosed with stage II breast cancer (67.7%) and received anthracycline‐based chemotherapy (69.1%).

**Table 1:** Baseline demographics and clinical characteristics of patents (n = 136)

Demographic information	Mean ± SD or Frequency (%)
Age (years)	51.5 ± 8.8
BMI (kg/m^2)^	23.9 ± 4.1
Ethnicity	
Chinese	112 (82.4)
Malay	12 (8.8)
Indian	7 (5.2)
Others^a^	5 (3.7)
Breast cancer stage	
I	15 (11.0)
II	92 (67.7)
III	29 (21.3)
ECOG Performance Status	
0	132 (97.1)
1	4 (2.9)
Baseline Haemoglobin levels	
<12 g/dL	35 (25.7)
>12 g/dL	101 (74.3)
Menopausal status	
Premenopausal	69 (50.7)
Postmenopausal	67 (49.3)
Chemotherapy Regimen	
Anthracycline‐based	94 (69.1)
Non‐anthracycline‐based	42 (30.9)
Baseline anxiety (BAI total score)^b^	7.25 ± 7.86
Baseline depression (BDI total score)^c^	6.48 ± 7.98
Baseline insomnia score^d^	22.3 ± 26.9

Abbreviation: ECOG as Eastern Cooperative Oncology Group.

a Others include: 4 Filipinos and 1 Sikh.

b BAI total score is 63 points.

c BDI total score is 63 points.

d Insomnia subscale total score is 100 points.

### 3.2 Prevalence of cancer‐related fatigue

Using the MCID as a cut‐off, patients who experienced a deterioration of ≥11 points from baseline were considered as fatigue cases. A total of 23.8% of patients experienced CRF at T3 compared to 13.8% of patients at T2, using the baseline as a reference. The overall incidence of CRF from T1 to T3 was 24.6%.

### 3.3 Plasma levels of adipokines and leptin across time points

The plasma levels of the individual adipokines are summarised as the medians with the interquartile ranges (Table 2). There were statistically significant changes in the plasma levels of leptin, IL‐6, Il‐8 and TNF‐α across the three time points (*P*‐value <0.001 using the Friedman test). Compared to baseline, a median increase of plasma leptin levels was observed at T2, followed by a decrease at T3 (T1: 4.07 ng/mL, T2: 4.95 ng/mL and T3: 3.96 ng/mL). For IL‐6, there was an increase in plasma levels towards the end of chemotherapy at T3 (T1: 0.80 pg/mL, T2: 1.11 pg/mL, T3: 1.27 pg/mL). For IL‐8, there was a decrease in plasma levels at T2, followed by an increase at T3 (T1: 3.70 pg/mL, T2: 3.33 pg/mL, T3: 3.69 pg/mL). For TNF‐α, there was a sustained increase in plasma levels (T1: 6.41 pg/mL, T2: 6.95 pg/mL, T3: 8.27 pg/mL).

**Table 2:** Comparison of plasma adipokine and leptin levels across the time points

Biomarker	Plasma adipokine concentration in Median (Inter‐quartile range)			Friedman Test	Post‐hoc analysis: Wilcoxon sign rank test		
	T1	T2	T3	*P*‐value	T1‐T2	T1‐T3	T2‐T3
Leptin (ng/mL)	4.07 (2.96, 6.61)	4.95 (3.27, 7.16)	3.96 (2.68, 6.07)	**<0.001**	**<0.001**	0.56	**<0.001**
IL‐6 (pg/mL)	0.80 (0.36, 1.72)	1.11 (0.57, 2.46)	1.27 (0.53, 2.60)	**<0.001**	**<0.001**	**<0.001**	0.07
IL‐8 (pg/mL)	3.70 (2.29, 4.95)	3.33 (2.25, 5.06)	3.69 (2.48, 5.28)	**<0.001**	0.42	**0.04**	**<0.001**
TNF‐α (pg/mL)	6.41 (2.76, 15.15)	6.95 (3.22, 18.61)	8.27 (2.71, 19.71)	**<0.001**	0.78	**<0.001**	**<0.001**

Abbreviation: TNF‐α, tumour necrosis factor‐α.

Bolded are *P*‐values < 0.05 and *P*‐values for post‐hoc analysis cut‐off = 0.0167.

In cases for patients whose plasma levels of adipokine were below the detection limit, the laboratory values are treated as missing values in the statistical analysis.

### 3.4 Correlation of plasma adipokines levels and confounders against plasma leptin levels at individual time points

Considering relationships between leptin and each individual adipokine, only IL‐8 had a statistically significant correlation at T1 (r = 0.20, *P* = 0.02). At T1, there were no significant correlations between leptin and IL‐6 or TNF‐α. At T2 and T3, none of the adipokines showed statistically significant correlations with leptin (Table 3).

**Table 3:** Correlation of plasma adipokine levels and known confounders against leptin at individual time points (T1, T2 and T3)

	T1		T2		T3	
	r	*P*‐value	r	*P* ‐value	r	*P* ‐value
IL‐6	0.14	0.18	0.07	0.50	0.07	0.52
IL‐8	**0.20**	**0.02**	0.06	0.46	0.06	0.46
TNF‐α	−0.03	0.71	−0.07	0.44	−0.02	0.85
Anxiety	−0.09	0.29	−0.03	0.70	0.008	0.92
Depression	**−0.20**	**0.02**	**−0.25**	**<0.001**	−0.08	0.37
Insomnia	−0.07	0.43	−0.02	0.83	**−0.20**	**0.02**
Body mass index	**0.59**	**<0.001**	**0.56**	**<0.001**	**0.58**	**<0.001**
Age	**0.23**	**<0.001**	0.15	0.08	0.16	0.07

Categorical variables	T1		T2		T3	
	**z‐score**	*P* ‐value	z‐score	*P* ‐value	z‐score	*P* ‐value
Menopausal status	−**3.14**	**0.002**	−1.87	0.06	−**2.51**	**0.01**
Anaemia status	−0.54	0.59	**2.34**	**0.02**	−0.46	0.64
Type of chemotherapy	0.44	0.66	−**2.14**	**0.03**	1.54	0.12

Abbreviation: TNF‐α, tumour necrosis factor‐α.

Bolded are *P*‐values < 0.05.

For continuous variables, data is presented as spearman correlation coefficients with r and its *P*‐values. For categorical variables, data is presented as the test statistic (z) along with p‐values of Mann‐Whitney U tests used to compare the proportion.

Among the confounders, leptin was positively correlated with BMI across all time points (T1: r = 0.59, T2: r = 0.56, T3: r = 0.58, all *P*‐values <0.001) and with age at T1 (r = 0.23, *P* < 0.001). There was a significant negative correlation between plasma leptin levels and depression at T1 and T2 (r = −0.20, *P* = 0.02 and r = −0.25, *P* < 0.001, respectively) and with insomnia at T3 (r = −0.20, *P* = 0.02). Among categorical variables, there was a difference in plasma leptin levels for menopausal status at T1 and T3 (*P* = 0.002 and 0.01 respectively), for anaemia status at T2 (*P* = 0.02) and the type of chemotherapy at T2 (*P* = 0.03).

### 3.5 Correlation of plasma adipokines levels, known confounders and leptin against fatigue levels across time points (T1‐T3)

There were consistent strong correlations with fatigue levels across all time points for anxiety, depression and insomnia. Age was negatively correlated with CRF across time points and CRF level also differed for the type of chemotherapy. There was only a significant negative correlation at T1 for leptin (r = −0.25, *P* = 0.005) (Table 4).

**Table 4:** Correlation of plasma adipokine levels, known confounders and leptin against fatigue levels across time points (T1‐T3)

	T1		T2		T3		T1‐T3	
	r	*P*‐value	r	*P*‐value	r	*P*‐value	*β* (SE)	*P*‐value
Leptin	−**0.25**	**0.005**	−0.11	0.22	0.02	0.83	−**0.56 (0.13)**	**<0.001**
IL‐6	0.007	0.94	0.17	0.10	0.09	0.36	0.50 (0.46)	0.27
IL‐8	−0.07	0.45	−0.004	0.96	0.04	0.65	0.10 (0.38)	0.12
TNF‐α	0.05	0.57	0.001	0.99	0.05	0.58	−**0.02 (0.004)**	**0.003**
Anxiety	**0.65**	**<0.001**	**0.63**	<**0.001**	**0.68**	<**0.001**	**1.38 (0.13)**	**<0.001**
Depression	**0.74**	**<0.001**	**0.74**	**<0.001**	**0.75**	<**0.001**	**1.85 (0.14)**	**<0.001**
Insomnia	**0.30**	**<0.001**	**0.34**	<**0.001**	**0.44**	<**0.001**	**0.15 (0.03)**	**<0.001**
Body mass index	−0.17	0.054	−0.05	0.59	−0.04	0.78	−0.39 (0.26)	0.13
Age	**−0.34**	**<0.001**	**−0.27**	**0.002**	−**0.25**	**0.004**	−**0.57 (0.12)**	**<0.001**

Categorical variables	T1		T2		T3		T1‐T3	
	z‐score	*P*‐value	z‐score	*P*‐value	z‐score	*P*‐value	*β* (SE)	*P*‐value
Menopausal status	1.78	0.08	1.11	0.27	1.49	0.14	−**5.59 (2.64)**	**0.04**
Anaemia status	−0.73	0.47	−0.43	0.67	**2.06**	**0.04**	0.02 (0.01)	0.09
Type of chemotherapy	**3.32**	**<0.001**	**3.57**	**<0.001**	**3.50**	**<0.001**	−**10.65 (2.32)**	**<0.001**

Abbreviation: TNF‐α, tumour necrosis factor‐α.

Bolded are p‐values < 0.05.

Across T1 to T3, leptin was negatively correlated with the total MFSI‐SF score (*β *= −0.56, *P* < 0.001), with every 1 unit decrease of leptin being associated with a 0.56 increase in total MFSI‐SF score (Table 2). TNF‐α was also inversely correlated with the total MFSI‐SF score (*β *= −0.02, *P* = 0.003). IL‐6 and IL‐8 did not show a statistically significant correlation with total MFSI‐SF score. For other individual explanatory variables, there were statistically significant positive associations for anxiety (*β* = 1.38, *P* < 0.001), depression (*β* = 1.85, *P* < 0.001) and insomnia (*β* = 0.15, *P* < 0.001) and significant negative correlations with menopausal status (*β* = −5.59, *P* = 0.04), age (*β *= −0.57, *P* < 0.001) and type of chemotherapy (*β* = −10.65, *P* < 0.001). Anaemia status (*β* = 0.02, *P* = 0.09) and BMI (*β *= −0.39, *P* = 0.13) were not significantly correlated with total MFSI‐SF score.

### 3.6 Adjusted model showing an association between leptin and CRF

Using *P*‐values <0.05 as selection criteria, the variables with statistically significant associations with CRF were TNF‐α, anxiety, depression, insomnia, age, menopausal status and type of chemotherapy. These variables were included as covariates in the final model. Results showed that leptin remained inversely associated with total MFSI‐SF score but the beta‐coefficient for leptin with the MFSI‐score was attenuated (*β *= −0.15, *P* = 0.003), with every 1 unit decrease of leptin being associated with a 0.14 increase in total MFSI‐SF score across time points (Table 5 **)**.

**Table 5:** Association of plasma leptin levels against total MFSI‐SF score after adjusting for statistically significant covariates

Variable	Total MFSI‐SF score	
	Coefficient *β* (SE)	*P*‐value
Constant	**19.80 (3.39)**	**<0.001**
Adjusted Leptin(ng/mL)	−**0.15 (0.05)**	**0.003**
TNF−α(pg/mL)	−**0.009 (0.002)**	**<0.001**
Anxiety	**0.74 (0.14)**	**<0.001**
Depression	**1.06 (0.17)**	**<0.001**
Insomnia	−0.002 (0.02)	0.94
Age	−**0.23 (0.08)**	**0.004**
Menopausal status	2.10 (1.50)	0.16
Type of chemotherapy	−1.75 (0.94)	0.06

Abbreviation: TNF‐α, tumour necrosis factor‐α.

Bolded are *P*‐values < 0.05.

While leptin was originally associated with the general (*β *= −0.16, *P* < 0.001), emotional (*β* = −0.11, *P* < 0.001) and mental sub‐domains (*β* = −0.06, *P* = 0.025) of MFSI‐SF scale, the beta coefficients of the respective five sub‐domains scores were not statistically significant in the final model (Table 6). In the analysis of other explanatory variables in the final model, there were positive significant associations with CRF for anxiety (*β* = 0.74, *P* < 0.001) and depression (*β* = 1.06, *P* < 0.001) and significant negative associations for TNF‐α (*β *= −0.009, *P* < 0.001) and age (*β* = −0.23, *P* = 0.004).

**Table 6:** Association of plasma leptin levels against total MFSI‐SF score and its respective sub‐domains after adjusting for statistically significant covariates

Variable (in ng/mL)	Total MFSI‐SF score			General		Physical		Emotional		Mental		Vigor	
	Coefficient *β* (SE)		*P*‐value	Coefficient *β* (SE)	*P*‐value	Coefficient *β* (SE)	*P*‐value	Coefficient *β* (SE)	*P*‐value	Coefficient *β* (SE)	*P*‐value	Coefficient *β* (SE)	*P*‐value
Constant	**19.80 (3.39)**		**<0.001**	**27.21 (1.41)**	**<0.001**	**25.57 (1.28)**	**<0.001**	**26.00 (1.07)**	**<0.001**	**25.55 (1.38)**	**<0.001**	**34.51 (2.71)**	**<0.001**
Adjusted Leptin	**−0.15 (0.05)**		**0.003**	−0.004 (0.04)	0.92	0.07 (0.04)	0.08	0.02 (0.03)	0.60	−0.005 (0.03)	0.90	0.12 (0.08)	0.13
TNF−α	**−0.009 (0.002)**		**<0.001**	0.002 (0.002)	0.34	−0.0008 (0.002)	0.68	0.0004 (0.002)	0.83	0.001 (0.002)	0.45	0.008 (0.004)	0.08
Anxiety	**0.74 (0.14)**		**<0.001**	**0.23 (0.03)**	**<0.001**	**0.21 (0.03)**	**<0.001**	**0.16 (0.02)**	**<0.001**	**0.11 (0.02)**	**<0.001**	**−0.11 (0.04)**	**0.004**
Depression	**1.06 (0.17)**		**<0.001**	**0.19 (0.04)**	**<0.001**	**0.12 (0.03)**	**<0.001**	**0.29 (0.03)**	**<0.001**	**0.16 (0.03)**	**<0.001**	**−0.31 (0.04)**	**<0.001**
Insomnia	−0.002 (0.02)		0.94	0.01 (0.006)	0.08	**0.01 (0.006)**	**0.04**	−0.004 (0.005)	0.44	0.006 (0.005)	0.22	−0.006 (0.009)	0.51
Age	**−0.23 (0.08)**		**0.004**	−0.05 (0.03)	0.14	**−0.07 (0.03)**	**0.03**	−0.04 (0.03)	0.08	−0.02 (0.03)	0.56	0.11 (0.06)	0.10
Menopausal status	2.10 (1.49)		0.16	0.61 (0.57)	0.29	**1.12 (0.50)**	**0.03**	0.43 (0.42)	0.30	−0.003 (0.58)	0.99	−1.22 (1.08)	0.26
Type of chemotherapy	−1.76 (0.94)		0.06	**−0.93 (0.40)**	**0.02**	0.09 (0.36)	0.81	−0.53 (0.30)	0.07	−0.25 (0.40)	0.53	**2.22 (0.82)**	**0.007**

Abbreviation: TNF‐α, tumour necrosis factor‐α.

Bolded are *P*‐values < 0.05.

## 4 DISCUSSION

In our cohort of early‐stage breast cancer patients, plasma leptin levels were found to be significantly and negatively correlated with total MFSI‐SF score across time points (*β* = −0.56, *P* < 0.01). This relationship remained significant (*β* = −0.15, *P* = 0.003) even after adjusting for the statistically significant covariates in our final model. This result does not support our initial hypothesis, in which we predicted that leptin levels would be positively correlated with fatigue levels, as observed in patient cohorts of other disease states.^6^, ^8^, ^9^

The difference in direction observed for the effect of leptin on fatigue suggests that the experience of CRF may be different from fatigue‐associated processes in other disease states. The positive correlations between fatigue and adipokines had been mostly observed in patients with other conditions marked by inflammation.^6^, ^9^, ^10^ For instance, in a cohort of patients with cardiovascular risk factors, plasma leptin levels were significantly and positively associated with fatigue score (r = 0.22, *P* < 0.001) and retained an independent association in a multiple logistic regression.^8^ Yet in another study evaluating patients with systemic lupus erythematosus, there was no significant association between adipokines and fatigue levels, but their correlation was reported to be negative. In that model, after adjustment for age, sex, race/ethnicity and BMI, leptin was negatively correlated with fatigue levels, measured using the Fatigue Severity Scale (*β* = −0.12, *P* = 0.61) and patient‐reported fatigue scores (*β* = −0.066, *P* = 0.96). The correlations between adipokines and both physical activity and disease activity were explained by differences in BMI.^32^

These observed differences in CRF may also be nuanced and subjected to changes that depend on the individual sub‐domains of CRF being examined. While CRF is a multidimensional construct, it is possible that not all facets of fatigue are equally associated with changes in adipokine levels. This is supported by our results in which plasma leptin levels were significantly correlated with the sub‐domains of general, emotional and mental fatigue but not the physical and vigour sub‐domains. In the final model, leptin was significantly correlated with only the total MFSI‐SF scores but not the remaining sub‐domains. This reinforces the need for studies to study the specific domains of fatigue more closely.

With regard to individual subdomains of fatigue, anxiety and depression continued to be strong predictors of fatigue levels over time, showing significant correlations with all five sub‐domains. This observation was also reported in a separate cohort of breast cancer survivors, in which the research team reported that patients diagnosed with depression and anxiety before chemotherapy were at higher risk of fatigue onset at a later period.^3^ This finding suggests that some psychological symptoms may form a fairly stable symptom cluster with CRF. It also supports the possibility that anxiety and depression add to the stress response and weaken the immune system, triggering inflammatory mediators involved in CRF and leading to pro‐inflammatory changes in the body.^33^ These findings are aligned with reviews reporting depression and anxiety as prominent correlates of CRF, 25, 33, 34 and lend strength to the argument that psychological distress needs to be accounted for when investigating the usefulness of leptin as a biomarker for CRF.

Based on our findings, it would be interesting to explore the relationships between leptin and other pro‐inflammatory biomarkers. Various cytokines such as TNF‐α, IL‐6 and IL‐1 can induce metabolic changes by mimicking the action of neuropeptides such as leptin because of their structural similarity.^35^ A decrease in food intake would usually suppress leptin expression, but in the presence of some cytokines, plasma leptin levels can be increased and work against the normal compensatory mechanisms set in place for leptin levels to regulate appetite. A possible explanation for the decrease in leptin levels with higher fatigue levels could be that an adaptive reduction of energy expenditure has been induced, resulting in increased appetite in response to metabolic impairment induced by cancer‐related inflammation. It might also be more meaningful to evaluate the ratio of plasma leptin to other known pro‐inflammatory cytokines to interpret inflammatory status, especially if they work as a network and if their levels affect each other. In the pathogenesis of some autoimmune diseases, leptin has been characterised together with adiponectin and resistin.^32^, ^36^ In an ovarian cancer study, pro‐inflammatory cytokines such as IL‐6 and TNF‐α were reported to induce an acute‐phase protein response that contributed to altered energy metabolism, which regulated leptin levels.^29^ In another cohort of lung cancer patients,^37^ the research team found significant positive associations among plasma levels of TNF‐α, IL‐1 and CRF. Together, these studies and our results suggest that leptin should be included in studies in which relationships between fatigue and cytokines are explored.

One strength of this study was the use of a longitudinal study design with repeated measurements of outcomes at various time points, which allowed us to determine whether a decrease in leptin levels signalled a rise in the fatigue experienced by breast cancer patients at a separate time point. Most of the current fatigue studies have been cross‐sectional so far,^8^, ^9^ and are not able to track the trajectories between fatigue and other variables, given how some of the other symptoms may be transient. A limitation of this study lies in that there were no cancer control patients who did not undergo chemotherapy to provide a comparison of CRF experience.

Understanding the role of leptin in relation to CRF may help us to devise more tailored interventions such as exercise to mitigate CRF in the future. In a randomised controlled trial examining the effects of exercise in overweight or obese breast cancer survivors,^38^ circulating biomarkers (insulin, IGF‐1, adiponectin and leptin) were significantly improved post‐intervention, compared to usual care. In another trial, a decrease in leptin concentrations in breast cancer survivors who exercised compared to controls was reported.^39^

Several concerns need to be addressed before any potential fatigue biomarker can have clinical utility. The observed difference in direction for effect of leptin on fatigue may be partly attributed to the non‐specific nature of fatigue, which can be challenging to quantify despite use of validated questionnaires and is dependent on the study tools used. There is no consistent definition of CRF and a general lack of agreement about which biomarkers to study or how to collect and test them. Research aimed at resolving these issues would help to advance the understanding of the mechanisms that underpin CRF and could improve the quality of life of individuals who experience it.

In conclusion, our data show that there is an inverse correlation between plasma leptin levels and fatigue levels over time in early‐stage breast cancer patients undergoing chemotherapy. The association with total score remained statistically significant after adjusting for known confounders of CRF, enabling leptin to function as a biomarker that could predict onset of CRF.

## CONFLICTS OF INTEREST

The authors confirm that there are no conflicts of interest.

## AUTHORS CONTRIBUTIONS

HHK, KO and AC: Conceptualisation, funding acquisition, supervision and writing‐review and editing. FKM and PC: investigation, project administration and resources. TCJ, AHLY, MS and GYX: data curation, investigation and project administration. TYL: Formal analysis, investigation, writing‐original draft, writing‐review and editing. All authors read and approved the final manuscript.
